# Understanding maternal decision-making for self-paid childhood vaccines a qualitative exploration of safety, information, and context

**DOI:** 10.3389/fpubh.2026.1849007

**Published:** 2026-08-24

**Authors:** Rong Xing, Yuanling Wu, Rui Liu, Manxia Hu, Yanli Lin, Liping Zhang, Yuting Hao, Junyan Zhang, Fang Gao

**Affiliations:** 1 Department of Preventive Healthcare, Shanxi Bethune Hospital, Shanxi Academy of Medical Sciences, Tongji Shanxi Hospital, Third Hospital of Shanxi Medical University, Taiyuan, China; 2 Department of Clinical Laboratory, Shanxi Bethune Hospital, Shanxi Academy of Medical Sciences, Tongji Shanxi Hospital, Third Hospital of Shanxi Medical University, Taiyuan, China; 3 Department of Pediatrics, Shanxi Bethune Hospital, Shanxi Academy of Medical Sciences, Tongji Shanxi Hospital, Third Hospital of Shanxi Medical University, Taiyuan, China; 4 Department of Obstetrics and Gynecology, Shanxi Bethune Hospital, Shanxi Academy of Medical Sciences, Tongji Shanxi Hospital, Third Hospital of Shanxi Medical University, Taiyuan, China; 5 Department of Preventive Healthcare, Xiaodian Community Health Service Center, Taiyuan, China; 6 Early Childhood Education Center, Shanxi Provincial Children’s Hospital, Taiyuan, China; 7 Evidence-Based Medicine, Shanxi Bethune Hospital, Shanxi Academy of Medical Sciences, Tongji Shanxi Hospital, Third Hospital of Shanxi Medical University, Taiyuan, China

**Keywords:** household context, maternal decision-making, non-National Immunization Program Vaccines, qualitative research, vaccine acceptance

## Abstract

**Objectives:**

To explore how mothers make decisions regarding self-paid childhood vaccines within real-world family and healthcare contexts, and to elucidate the underlying processes that may explain variability in maternal willingness observed in prior quantitative findings.

**Methods:**

A qualitative study was conducted using semi-structured, in-depth interviews among biological mothers of children aged 42 days to 3 years attending a preventive healthcare outpatient clinic. Participants were purposively sampled based on prior vaccination experience and willingness to capture diverse decision-making patterns across predefined groups. Interviews were audio-recorded, transcribed verbatim, and analyzed using thematic analysis with inductive coding. Data collection proceeded iteratively until thematic saturation was achieved.

**Results:**

A total of 16 mothers participated. Five themes and fifteen subthemes were identified, capturing key dimensions of maternal decision-making. Mothers navigated multiple and asymmetric information sources, balancing trust in healthcare professionals with independent verification or reliance on social networks. Decision-making followed a safety-first structure, in which perceived safety functioned as a prerequisite before consideration of benefits. Economic considerations were framed as a dynamic tension between immediate financial burden and perceived long-term health value, with cost acting as either a filtering factor or a selective prioritization mechanism. Trust in healthcare providers was constructed through communication quality, while insufficient consultation contributed to uncertainty and increased reliance on informal information sources. System-level factors, including service convenience, digital accessibility, and lack of follow-up support, further shaped decision-making processes.

**Conclusion:**

Maternal decision-making regarding self-paid childhood vaccines is a dynamic and context-dependent process characterized by safety-first appraisal, information uncertainty, economic evaluation, and communication experiences. Family context influences how these factors are prioritized and negotiated. Strategies to improve uptake should address safety concerns explicitly, enhance accessible information delivery, and strengthen communication and continuity within vaccination services.

## Background

Vaccination remains a cornerstone of child health protection (1), yet not all recommended vaccines are incorporated into national immunization schedules. Non-National Immunization Program vaccines (non-NIPV), which are recommended but not publicly funded, occupy a distinct position within childhood vaccination systems. Decisions regarding these vaccines are characterized by discretionary choice, out-of-pocket payment, and the absence of uniform policy mandate, requiring parents to actively evaluate potential benefits, risks, and costs rather than simply follow routine schedules (2). In this context, maternal decision-making plays a central role, particularly during early childhood when vaccination encounters are frequent and health-related judgments are made under conditions of uncertainty (3).

In the quantitative phase of this study, variability in maternal willingness to vaccinate children with non-NIPV was observed across different household contexts. Patterns of willingness were not uniform, suggesting that family circumstances may be associated with how mothers approach discretionary vaccination decisions (4). These findings indicated the presence of structured differences rather than random variation, pointing to underlying decision-making processes that extend beyond individual attitudes captured by questionnaire-based measures (5).

However, the quantitative phase of this study was not designed to explore the internal decision-making processes underlying maternal willingness toward non-NIPV. While questionnaire-based analyses can describe patterns of willingness, they cannot capture how mothers interpret vaccination information, appraise safety and benefit, or navigate uncertainty within their family context. Consequently, the mechanisms through which observed quantitative patterns arise remain insufficiently explained by survey data alone.

To address this gap, a qualitative study was undertaken to explore how mothers make decisions regarding non-NIPV within their everyday family and healthcare contexts. By focusing on maternal perspectives and decision-making processes, this qualitative inquiry was intended to provide interpretive depth to the quantitative findings and to clarify how observed patterns of willingness are formed.

## Methods

The study was conducted at a tertiary hospital in Shanxi Province serving both urban and surrounding rural populations. In this setting, both NIPV and non-NIPV are routinely available through community vaccination clinics, although coverage of non-NIPV varies across vaccine types and remains lower than that of NIPV.

### Study design

This study employed a qualitative research design using semi-structured, in-depth interviews to explore maternal decision-making processes regarding non-NIPV. The qualitative approach was chosen to gain an in-depth understanding of how mothers perceive, interpret, and negotiate vaccination decisions in contexts where vaccines are recommended but not publicly funded. The study was interpretive in nature and aimed to capture participants’ experiences, perceptions, and reasoning rather than to quantify associations or test hypotheses.

### Participants and setting

Participants were biological mothers aged 18 years or older with at least one child aged between 42 days and 3 years who attended routine child healthcare outpatient services at the Department of Preventive Healthcare, Shanxi Bethune Hospital. The interview sample was drawn from mothers who had previously participated in a questionnaire survey on childhood vaccination decision-making and had agreed to be contacted for further interviews. Mothers were required to provide written informed consent prior to participation.

Participants were excluded if their child had congenital or genetic diseases or primary immunodeficiency disorders, if the mother had difficulties in comprehension or communication that precluded participation in an interview, if the child had already received all available non-NIPV, or if the mother or child was participating in other clinical studies that could influence vaccination-related decision-making.

### Sampling strategy

A purposive sampling strategy was adopted to ensure variation in maternal experiences and perspectives related to non-NIPV decision-making. Participants were selected based on their expressed willingness to vaccinate and prior non-NIPV uptake, with the aim of capturing diverse decision-making patterns rather than achieving statistical representativeness. Sampling was iteratively refined as data collection progressed, and interviews continued until information saturation was achieved (6), defined as the point successive interviews generated no new substantive themes and only repeated concepts identified in earlier interviews. Based on subgroup characteristics identified during preliminary screening with respect to willingness to vaccinate, four typical groups were purposively sampled:

1. Group 1 (G1): willing to vaccinate with non-NIPV without prior non-NIPV experience (3 to 5 participants)
2. Group 2 (G2): unwilling to vaccinate with non-NIPV, despite prior non-NIPV experience (3 to 5 participants)
3. Group 3 (G3): hesitant primarily due to safety concerns (3 to 5 participants)
4. Group 4 (G4): hesitant primarily due to cost considerations (3 to 5 participants)

To facilitate comparison across family contexts, participants were drawn from both single-child households (SCH) and multiple-children households (MCH), defined as families with two or more children.

Participants were purposively recruited from four predefined groups to ensure variation in vaccination attitudes and experiences. These predefined groups were used solely to guide participant recruitment rather than data analysis. Interview transcripts from all participants were analyzed together using an inductive thematic analysis approach, from which themes were generated. Recruitment and analysis proceeded iteratively, and interviews continued until thematic saturation was achieved across the dataset. The final sample comprised 16 participants, with four participants in each group.

### Data collection

Interviews were conducted by two female researchers, both holding master’s degrees, with experience in preventive healthcare and qualitative interviewing. Before data collection, both interviewers received standardized training in qualitative interviewing techniques and use of the semi-structured interview guide. The interviewers had no prior personal relationship with the participants other than their role in the research, and participants were informed of the study objectives before the interviews. No repeat interviews were conducted.

Qualitative data were collected through one-to-one, face-to-face semi-structured interviews conducted by trained researchers. An interview guide was developed based on existing literature and clinical experience and was used flexibly to allow participants to elaborate on issues they considered most salient. Interview topics included mothers’ understanding of non-NIPV, sources and evaluation of vaccination information, perceptions of vaccine safety and disease-related risks, trust in healthcare professionals and the healthcare system, intra-family discussions and negotiations, financial considerations related to vaccine costs, and experiences with vaccination services.

Interviews lasted approximately 40 min on average and were audio-recorded with participants’ consent. All recordings were transcribed verbatim. Transcripts and findings were not returned to participants for member checking. Field notes were taken during and immediately after interviews to capture contextual information and preliminary analytic reflections. An interview guide, developed based on existing literature and clinical experience, is presented in Table 1.

**Table 1:** Semi-structured interview questions.

No.	Interview Questions
1	When choosing vaccines for your child, including both NIPV and non-NIPV, what sources do you primarily rely on for information? How reliable and comprehensive do you consider these sources to be?
2	When you hear the term “non-NIPV” (self-paid vaccines), what comes to mind first? What do you perceive as the main differences between these vaccines and those included in the NIP?
3	Vaccine safety is a common concern among parents. What are your personal views on the safety of non-NIPV? Have you had any specific concerns, and if so, where did these concerns originate? How do you usually respond when such concerns arise?
4	When deciding whether to vaccinate your child with a non-NIPV, what factors do you usually consider (for example, cost, effectiveness in preventing disease, disease severity, your child’s physical condition, or convenience of vaccination)? What role does cost play in your decision-making?
5	During the decision-making process, have you experienced internal hesitation or differences of opinion within your family? How did you manage these differences and arrive at a final decision?
6	In your view, what could healthcare institutions, communities, or relevant authorities do to help parents feel more confident and supported when deciding on non-NIPV for their children? If you could offer one most important suggestion, what would it be?

Although all participants were initially asked the same broad, open-ended questions, interviewers used flexible follow-up probes to further explore participants’ vaccination attitudes, previous experiences, and the reasons underlying their decision-making according to issues emerging during each interview.

### Data analysis

Interview transcripts were analyzed using thematic analysis. Analysis proceeded iteratively and involved repeated reading of transcripts to achieve immersion in the data. Initial codes were generated inductively from the data and were subsequently organized into broader themes that reflected recurring patterns in participants’ accounts. Two researchers independently coded the interview transcripts using an inductive approach. Coding discrepancies were discussed until consensus was reached, with unresolved issues reviewed and adjudicated by the primary corresponding author. No predefined coding framework was established before data analysis. Instead, codes were developed inductively from the interview transcripts and were continuously refined through iterative discussion as themes emerged during the analytic process. No qualitative data analysis software was used. Data management and coding were conducted manually throughout the analytic process.

Throughout the analytic process, attention was paid to similarities and differences across participants from different household contexts, while avoiding *a priori* assumptions about the direction or magnitude of such differences. Data management and organization were conducted manually and using standard qualitative analysis procedures.

Themes were developed inductively from the interview data and were not mapped directly onto individual interview questions; rather, they reflected patterns emerging across multiple questions and participants.

### Methodological rigor

To support methodological rigor, several additional qualitative research practices were applied. These included verbatim transcription of interviews, iterative comparison of data during analysis, and regular team discussions during coding and theme development.

## Results

### Participants’ characteristics

A total of 16 biological mothers participated in the in-depth interviews. Participants ranged in age from 27 to 40 years and represented diverse educational backgrounds, spanning from senior high school to postgraduate training. The sample included mothers from both SCH and MCH, allowing coverage of family structures relevant to non-NIPV decision-making contexts. The interviewed children covered key early-life immunization decision windows, with ages ranging from 2 months to 2 years at the time of interview. Detailed characteristics of interview participants are presented in Table 2.

**Table 2:** Characteristics of participants.

Participant ID	Group	Age	Education level	Occupation	Household monthly income (RMB)	Residence	Child sex	Preterm birth	Gravida para	Birth order of interviewed child	Child age at interview	With siblings
F1	G1	29	Bachelor’s degree	Full-time mother	5,001–10,000	Urban	Female	No	G1P1	Only child	3 months	None
F2	G3	30	Senior high school	Occupation not reported	Below 3,000	Urban	Male	No	G1P1	Only child	9 months	None
F3	G4	36	Master’s degree	Company employee	More than 20,000	Urban	Male	No	G2P2	Second-born	8 months	Older sister
F4	G1	29	Junior college	Company employee	3,000–5,000	Urban	Female	No	G1P1	Only child	7.2 months	None
F5	G4	32	Bachelor’s degree	Civil servant	5,001–10,000	Urban	Female	No	G2P2	Second-born	5.3 months	Older brother
F6	G2	30	Senior high school	Company employee	3,000–5,000	County	Male	No	G2P2	Second-born	18 months	Older brother
F7	G3	40	Medical master’s degree	Physician	10,001–20,000	Urban	Female	No	G1P1	Only child	5.4 months	None
F8	G2	30	Junior college	Company employee	3,000–5,000	Urban	Male	No	G1P1	Only child	5.1 months	None
F9	G4	32	Bachelor’s degree	Teacher	5,001–10,000	Urban	Male	No	G2P2	First-born	2 years	Younger sister
F10	G1	27	Senior high school	Full-time mother	Below 3,000	County	Male	No	G1P1	Only child	6.1 months	None
F11	G3	35	Master’s degree	Civil servant	10,001–20,000	Township	Female	Yes	G2P2	Second-born	4.1 months	Older sister
F12	G2	29	Bachelor’s degree	Civil servant	5,001–10,000	Township	Male	No	G1P1	Only child	2 months	None
F13	G1	33	Bachelor’s degree	Company employee	3,000–5,000	Urban	Female	No	G1P1	Only child	10.4 months	None
F14	G2	34	Bachelor’s degree	Occupation not reported	10,001–20,000	Urban	Male	No	G2P2	Second-born	3.4 months	Older sister
F15	G3	32	Master’s degree	Self-employed	More than 20,000	County	Male	No	G1P1	Only child	11 months	None
F16	G4	27	Bachelor’s degree	Company employee	5,001–10,000	Urban	Female	No	G1P1	Only child	1 year	None

Group 1 (G1): willing to vaccinate with non-NIPV without prior non-NIPV experience.

Group 2 (G2): unwilling to vaccinate with non-NIPV, despite prior non-NIPV experience.

Group 3 (G3): hesitant primarily due to safety concerns.

Group 4 (G4): hesitant primarily due to cost considerations.

Participants were distributed evenly across the four predefined analytic groups, with four participants in each group (G1–G4). Across these groups, variation was observed in prior vaccination experience, current willingness, and dominant sources of hesitation. Notably, one participant (F7) had received postgraduate-level medical training, providing a perspective informed by professional medical knowledge. Although concerns related to safety and cost were more frequently articulated among mothers from MCH, such concerns were also observed across SCH and MCH, underscoring the diversity of maternal decision-making contexts represented in the sample.

### Overview of themes

Five themes and fifteen subthemes emerged from the qualitative analysis, illustrating mothers’ experiences with information seeking, risk–benefit appraisal, financial considerations, healthcare communication, and service-related expectations in relation to non-NIPV (Table 3).

**Table 3:** Themes and subthemes derived from qualitative interviews.

Theme	Subthemes
1. Multiplicity and asymmetry of information sources	1.1 Coexistence of reliance on professional authority and active verification
	1.2 Dual effects of social networks and experiential information sharing
	1.3 Information presentation and barriers to comprehension
2. Safety–benefit trade-offs in vaccination decision-making	2.1 Safety as the primary consideration
	2.2 Assessment of disease burden and preventive effectiveness
	2.3 Consideration of child-specific health characteristics
3. Tension between economic burden and health investment	3.1 Cost as a filtering factor or decisive barrier
	3.2 Cost–effectiveness evaluation from a health investment perspective
	3.3 Expectations regarding insurance coverage and social support
4. Trust building and gaps in healthcare communication	4.1 Characteristics of high-quality communication: empathy, explanation, and time
	4.2 Perceived insufficiency of communication in routine vaccination settings
	4.3 Trust grounded in professional competence and transparency
5. System-level support and individual expectations in service experience	5.1 Convenience and human-centered vaccination procedures
	5.2 Demand–gap mismatch in digital vaccination services
	5.3 Expectations for continuous and follow-up support

### Theme 1. Multiplicity and asymmetry of information sources

Mothers described accessing vaccination-related information through multiple channels, with varying degrees of trust and perceived reliability. Information sources ranged from healthcare professionals and official medical platforms to social media and peer networks, often resulting in uneven information quality and informational asymmetry.

#### Subtheme 1.1 coexistence of reliance on professional authority and active verification

Across participants, healthcare professionals were consistently regarded as the most authoritative and trusted source of vaccination-related information. Many mothers described physicians’ explanations as central to clarifying vaccine indications, disease severity, and potential adverse reactions, particularly when decisions involved non-NIPV. One mother noted, “The most important thing for me was still the advice from the child healthcare doctor” (F1).

However, the extent to which medical advice was accepted passively or actively verified varied markedly. Mothers with higher educational attainment or medical-related backgrounds tended to engage in independent verification and evidence-based comparison. For example, a participant with a medical background reported consulting professional literature and clinical trial data when selecting vaccines: “I compared the clinical trial data of different rotavirus vaccines, including efficacy and serotype coverage, before choosing the pentavalent one” (F7). Similarly, participants with research experience described cross-referencing physicians’ recommendations with international guidelines and peer-reviewed evidence before finalizing decisions.

In contrast, mothers with lower educational attainment or limited digital literacy reported relying almost exclusively on physicians’ brief recommendations, often without sufficient opportunity for clarification. One participant explained, “The doctor just asked, ‘This one is self-paid, do you want it?’ I did not really understand what it was for, and there wasn’t time to ask more” (F2). For these mothers, trust in professional authority coexisted with a sense of uncertainty and informational insufficiency.

#### Subtheme 1.2 dual effects of social networks and experiential information sharing

Social networks, including parenting groups, relatives, and peers with childcare experience, functioned as important supplementary information sources, particularly for mothers who felt less confident navigating online medical information. Several participants described relying on friends, family members, or childcare workers to interpret vaccine-related risks and post-vaccination reactions. One mother stated, “I mostly asked relatives and colleagues who already had children. Their experience felt the most reliable to me” (F2).

At the same time, participants frequently described social media and group chats as sources of information overload and anxiety. Exposure to fragmented or emotionally charged narratives, especially reports of adverse reactions, amplified uncertainty rather than resolving it. As one participant noted, “Every day there is new information saying different things, and I really do not know whom to trust” (F12). This effect was particularly pronounced among mothers who lacked the confidence or skills to independently evaluate the credibility of shared information.

#### Subtheme 1.3 information presentation and barriers to comprehension

Participants expressed strong preferences for information presented in intuitive, accessible formats, such as short videos, visual aids, and plain-language explanations. Mothers with limited formal education or medical knowledge reported difficulty understanding official written materials, which were often perceived as overly technical. One participant remarked that “the information on official leaflets is hard to understand, but short videos explain things much more clearly” (F4).

For these mothers, comprehension barriers reduced their ability to actively engage in decision-making, reinforcing dependence on simplified messages or interpersonal guidance. In contrast, participants with higher educational backgrounds emphasized the value of detailed data, technical explanations, and transparent risk communication, highlighting divergent informational needs within the same vaccination system.

### Theme 2. Safety-benefit trade-offs in vaccination decision-making

Across interviews, mothers consistently described decision-making regarding non-NIPV as a process of balancing perceived safety against anticipated health benefits. Although safety concerns were universally salient, the way in which safety and benefit were interpreted, prioritized, and weighed varied markedly across participants, reflecting differences in health literacy, prior experiences, and child-specific health conditions.

#### Subtheme 2.1 safety as a non-negotiable prerequisite in decision-making

For many mothers, safety functioned as a prerequisite for considering non-NIPV, rather than one dimension within a broader trade-off. Concerns commonly centered on severe adverse reactions, vaccine quality, and uncertainties related to manufacturing and transportation processes. Mothers with lower confidence in interpreting medical information often described safety in general and undifferentiated terms, expressing difficulty in judging risks beyond a basic sense of unease. One participant noted, “I worry whether the vaccine quality is really guaranteed, especially during transportation. This kind of thing is impossible for us to check ourselves” (F2).

In contrast, some mothers articulated safety in more specific and structured ways, framing risk as probabilistic and regulated rather than absolute. These participants emphasized regulatory oversight, post-marketing surveillance, and comparative risk between vaccination and disease. One mother explained, “Any vaccine has a very small risk, but compared with the disease itself, vaccination is much safer. Non-NIPV follow the same approval standards” (F3). Despite these differences in framing, safety remained the initial threshold that had to be satisfied before other considerations could enter decision-making.

Child health history further intensified safety salience. For a mother whose child was born prematurely, safety appraisal was particularly cautious and detailed. She described heightened attention to vaccine quality control and potential biological effects, attributing this vigilance to concerns related to her child’s early birth (F11). Importantly, this heightened sensitivity did not translate into outright refusal but rather into more deliberate evaluation.

#### Subtheme 2.2 assessment of disease severity and preventive benefit

Beyond safety, mothers evaluated the perceived severity of vaccine-preventable diseases and the expected effectiveness of vaccination. Diseases associated with hospitalization, severe symptoms, or visible suffering were more likely to motivate vaccination. Several mothers referenced personal or indirect experiences as key triggers. One participant recalled, “My nephew was hospitalized due to rotavirus infection. Seeing such a real and severe case made me feel that this was something my own child should never have to go through” (F1).

Mothers with higher confidence in understanding health information tended to assess benefit through comparisons of effectiveness, duration of protection, and scope of disease prevention. For example, one participant described comparing protection profiles across different vaccine options before making a decision (F7). In contrast, mothers who felt less able to access or interpret formal evidence relied more heavily on disease visibility within their social environment, such as reports of outbreaks in childcare settings, to judge whether vaccination was worthwhile (F4, F8).

For the mother of a preterm child, perceived benefit was closely linked to vulnerability. She described vaccination as a preventive measure to reduce the risk of severe infection, given her child’s increased susceptibility, while simultaneously emphasizing the importance of well-established safety profiles (F11). This dual focus highlighted how child-specific factors could amplify both sides of the safety–benefit evaluation.

#### Subtheme 2.3 role of child-specific health characteristics in shaping decisions

Individual child characteristics played a central role in shaping how safety and benefit were weighed. Mothers frequently mentioned allergies, physical constitution, or previous illnesses as factors requiring professional clarification. Clear guidance from healthcare providers regarding contraindications and post-vaccination observation was often described as reassuring. One participant stated, “My child has mild eczema. The doctor explained clearly that it was not a contraindication and told me what to watch for afterward, which made me feel at ease” (F5).

However, when child-specific concerns intersected with limited information access or external pressures, decision-making became more constrained. Some mothers described delaying or foregoing vaccination due to unresolved safety concerns, family disagreement, or financial considerations, even when potential benefits were acknowledged (F6). These accounts illustrated how safety–benefit evaluations were embedded within broader personal and contextual constraints rather than determined by medical considerations alone.

### Theme 3. Tension between economic burden and health investment

Mothers commonly described economic considerations as an unavoidable component of decision-making regarding non-NIPV. Rather than viewing cost as a simple barrier, participants framed payment decisions through a dynamic tension between immediate financial burden and perceived long-term health value. How this tension was resolved varied across families, shaped by household resources, perceptions of value, and competing family priorities.

#### Subtheme 3.1 cost as a filtering factor or decisive barrier

For some mothers, vaccine cost functioned as an initial filter that determined whether non-NIPV entered consideration at all. When household budgets were constrained or vaccination expenses coincided with other major family expenditures, cost was described as a decisive barrier. One participant stated, “If it’s too expensive, I will not even think about it. There are too many things to spend money on when raising a child” (F2). Another mother explained that even when she acknowledged the potential benefits of vaccination, the out-of-pocket price made it difficult to proceed (F6).

In these accounts, economic burden was not framed as opposition to vaccination itself but as a practical limitation. Mothers emphasized that the absence of public funding or reimbursement placed the full financial responsibility on families, making non-NIPV decisions more sensitive to short-term affordability.

#### Subtheme 3.2 evaluating vaccination as a health investment

In contrast, some mothers described approaching non-NIPV through a health investment perspective, weighing cost against anticipated reductions in future medical expenses, caregiving burden, and emotional stress. These mothers articulated willingness to pay higher upfront costs when vaccination was perceived to prevent severe illness or hospitalization. One participant noted, “Spending money now can save much more trouble later, especially if it prevents serious disease” (F7).

This investment-oriented framing was often accompanied by selective prioritization. Rather than accepting all self-paid vaccines, mothers described choosing specific vaccines perceived as offering higher value. One participant explained that she evaluated “which vaccines are really necessary and which ones can be postponed,” indicating an active process of prioritization rather than blanket acceptance or rejection (F1).

#### Subtheme 3.3 expectations regarding insurance coverage and institutional support

Across interviews, mothers expressed strong expectations for greater institutional involvement in reducing the financial burden associated with non-NIPV. Many participants questioned why vaccines perceived as important for child health required full out-of-pocket payment. One mother remarked, “If this vaccine is truly important, why does the family have to pay everything themselves?” (F4).

Some participants expressed hope that insurance coverage, partial subsidies, or future inclusion in public programs could alter their decisions. Others described feeling that the current payment model shifted responsibility entirely to families, intensifying hesitation and moral pressure around decision-making. These expectations highlighted how economic considerations were closely intertwined with perceptions of policy endorsement and institutional responsibility, rather than functioning as purely individual financial calculations.

### Theme 4. Trust building and gaps in healthcare communication

Mothers consistently described trust in healthcare providers as central to their vaccination decisions. However, trust was not treated as a fixed attribute of the healthcare system; instead, it was constructed through concrete communication experiences. When communication aligned with mothers’ informational and emotional needs, trust was reinforced. Conversely, when interactions were brief, unclear, or dismissive, trust was weakened, even when professional authority was not explicitly questioned.

#### Subtheme 4.1 characteristics of communication that foster trust

High-quality communication was commonly described in terms of clarity, patience, and responsiveness. Mothers valued explanations that went beyond simple recommendations and addressed practical concerns, such as how vaccines work, what reactions to expect, and how to manage potential adverse events. One participant noted that detailed explanations helped her feel reassured and confident in her decision, stating that “when the doctor explained clearly what might happen after vaccination and what signs to watch for, I felt much more at ease” (F5).

Time and attitude were also emphasized as critical components of trust-building. Mothers described feeling respected when healthcare providers allowed questions and responded without impatience. In these encounters, trust was grounded not only in professional expertise but also in the perception that providers were attentive to individual concerns rather than delivering standardized advice.

#### Subtheme 4.2 perceived insufficiency of communication in routine vaccination settings

In contrast, many mothers described routine vaccination encounters as rushed and transactional. Limited consultation time and high patient volume were frequently cited as barriers to meaningful communication. Several participants reported that recommendations regarding non-NIPV were delivered briefly, sometimes framed as optional add-ons, with little accompanying explanation. One mother recalled, “The doctor just said it’s self-paid and asked whether we wanted it. There wasn’t much explanation, and the line behind us was very long” (F2).

These communication gaps did not necessarily lead to overt mistrust of healthcare professionals but contributed to hesitation and reliance on alternative information sources. Mothers described leaving vaccination clinics with unresolved questions, which were later addressed through social networks or online searches. This shift increased exposure to inconsistent information and, in some cases, heightened anxiety rather than resolving uncertainty.

#### Subtheme 4.3 trust grounded in perceived professionalism and transparency

Despite communication constraints, trust was often maintained when mothers perceived healthcare providers as transparent and professionally consistent. Clear acknowledgment of uncertainties, balanced discussion of benefits and risks, and avoidance of overly directive language were described as enhancing credibility. One participant expressed appreciation when a provider openly discussed both advantages and limitations of vaccination options, noting that “being honest about risks made the advice feel more reliable” (F7).

Mothers also emphasized continuity and consistency across encounters. Repeated interactions with the same providers or alignment between information received from different healthcare settings strengthened trust. Conversely, inconsistent messages or perceived reluctance to discuss non-NIPV in depth were described as undermining confidence, even when professional qualifications were not questioned.

### Theme 5. System-level support and service experience in vaccination

Beyond individual considerations, mothers’ vaccination decisions were shaped by their experiences with the broader vaccination service system. Service organization, procedural convenience, and continuity of support influenced not only practical feasibility but also mothers’ confidence in navigating non-NIPV decisions. These system-level factors interacted with individual concerns, either facilitating or constraining uptake.

#### Subtheme 5.1 convenience and human-centered vaccination procedures

Mothers frequently described convenience as a practical enabler of vaccination decisions. Short waiting times, clear procedures, and child-friendly environments were perceived as reducing stress for both parents and children. Some participants noted that when the vaccination process was smooth and orderly, they were more willing to consider additional vaccines beyond those included in the routine schedule. One mother explained that “when everything is well arranged and not too rushed, it feels easier to accept extra vaccines” (F8).

Conversely, overcrowded clinics and rigid procedures were described as discouraging, particularly for mothers managing young children. Several participants emphasized that long queues and limited flexibility made it difficult to ask questions or reflect on decisions, reinforcing a tendency to postpone or decline non-NIPV despite recognizing potential benefits (F2, F6).

#### Subtheme 5.2 mismatch between digital services and user needs

Digital tools, such as online appointment systems and electronic vaccination records, were widely recognized as convenient in principle. However, mothers reported uneven experiences in practice. While some participants appreciated the efficiency of digital platforms, others described difficulties navigating interfaces or accessing reliable information through official channels. One mother remarked that “the app is useful for booking, but it does not really help me understand which vaccines are important or why” (F4).

For mothers with limited digital literacy, reliance on online systems sometimes increased confusion rather than reducing it. In these cases, the absence of alternative support pathways, such as in-person guidance or follow-up explanations, amplified uncertainty and delayed decision-making (F10).

Another participant appreciated the convenience of digital vaccination services, noting that “online appointment systems save us a lot of time and effort, and electronic vaccination records make it much easier to check my child’s vaccination history. I also hope the system could provide reminders before scheduled vaccinations” (F13).

#### Subtheme 5.3 expectations for continuity and follow-up support

Across interviews, mothers expressed a desire for greater continuity in vaccination-related support. Rather than one-time encounters, participants described valuing ongoing access to guidance, particularly when concerns arose after vaccination or when decisions involved multiple doses over time. One participant noted that “after vaccination, there is no clear channel to ask questions if something worries you later” (F5).

Similar views were expressed by another participant, who suggested that “appointment-based, one-to-one consultation would allow doctors sufficient time to explain vaccines in language that parents can understand, which would further increase my confidence in vaccination” (F16).

Some mothers suggested that follow-up communication, reminders, or opportunities for subsequent consultation could strengthen confidence and reduce reliance on informal information sources. These expectations highlighted a perceived gap between the complexity of non-NIPV decision-making and the episodic nature of current vaccination services.

## Discussion

In childhood immunization, mothers commonly serve as the primary caregivers and principal decision-makers, particularly during early life stages when vaccination schedules are most intensive (7). Their perceptions, experiences, and evaluations therefore play a central role in shaping vaccination uptake, especially in contexts where vaccines are recommended but not publicly funded (3). Unlike vaccines included in national immunization programs, decisions regarding non-NIPV require active parental deliberation (4), as they involve discretionary choice, out-of-pocket payment, and the absence of uniform policy endorsement. Understanding maternal decision-making in this context thus requires attention not only to whether mothers accept or decline vaccination, but also to how they interpret information, assess risks and benefits, negotiate financial considerations, and interact with healthcare services.

### Principal interpretive findings

The conceptual terms used in this discussion represent the research team’s interpretive synthesis of participants’ experiences and were developed inductively from recurring patterns identified across the interview data. This qualitative study suggests that maternal decision-making regarding non-NIPV is not driven by a single determinant, but rather reflects a dynamic process in which multiple considerations are weighed under conditions of informational uncertainty and discretionary choice (8). Across the five themes, mothers described navigating asymmetric information environments, safety thresholds, economic trade-offs, communication experiences, and system-level service constraints when considering non-NIPV.

Importantly, although family context is not the sole factor determining vaccination decisions (9), it provided an important perspective for understanding maternal decision-making in this study. Mothers from single-child and multiple-child households described the same overarching themes related to information, safety, economic considerations, healthcare communication, and service experience. Household context primarily influenced how these shared themes were interpreted, prioritized, and translated into vaccination decisions. Previous parenting experience, family responsibilities, and financial commitments shaped the relative importance of specific concerns within a common decision-making framework. These findings suggest that household structure functions primarily as a contextual factor influencing the expression of shared decision-making processes rather than producing fundamentally different decision-making processes.

### Interpretation of theme 1: information multiplicity and asymmetry

Theme 1 indicates that maternal decision-making regarding non-NIPV occurs within an information environment characterized by multiple sources and pronounced asymmetry in credibility and comprehensibility. Mothers accessed information from healthcare professionals, official platforms, social media, and peer networks; however, the coexistence of these channels often intensified uncertainty rather than resolving it, particularly when messages were fragmented or inconsistent.

Healthcare professionals were consistently regarded as the most trusted source, yet trust did not necessarily imply passive acceptance. Mothers varied in their ability and willingness to independently verify medical advice, leading to differential interpretation and use of identical clinical messages. Social networks functioned as supplementary sources when formal communication was perceived as insufficient, but emotionally salient narratives, especially those emphasizing adverse events, frequently amplified risk perceptions. In addition, mismatches between information formats and mothers’ comprehension needs further contributed to asymmetry. Official materials were often viewed as technically accurate but difficult to understand, whereas simplified formats were perceived as more accessible.

Overall, non-NIPV decisions appear to be shaped within an uneven information ecology, in which information structure acts as an upstream influence on subsequent evaluations of safety, benefit, and cost. Given that healthcare professionals remain the most trusted source of information, while mothers vary substantially in their capacity to interpret medical content, there is a clear need for more effective and diversified health communication strategies. In particular, translating professionally endorsed information into accessible formats and disseminating it through commonly used social media platforms may help bridge existing comprehension gaps and reduce reliance on fragmented or emotionally driven information sources.

### Interpretation of theme 2: safety-benefit appraisal

Theme 2 illustrates that maternal evaluation of non-NIPV is structured less as a linear weighing of risks and benefits than as a threshold-based appraisal process. Across interviews, safety was consistently described as a prerequisite for consideration, rather than one dimension to be traded against anticipated benefits. For many mothers, concerns regarding vaccine quality, adverse reactions, and regulatory oversight had to be sufficiently addressed before any discussion of disease prevention or effectiveness could meaningfully proceed.

Within this framework, perceptions of disease severity and preventive benefit became salient only after safety concerns were perceived as manageable (10). Mothers often relied on concrete or emotionally salient experiences, such as hospitalization of relatives’ children or outbreaks within familiar social environments, to anchor their assessment of disease risk. These patterns suggest that benefit appraisal in non-NIPV decisions is closely tied to experiential salience rather than abstract reasoning alone (11).

Child-specific health characteristics further shaped this appraisal process. Rather than uniformly discouraging vaccination, factors such as prematurity or minor chronic conditions heightened mothers’ demand for clear, individualized explanations. When professional guidance successfully contextualized these characteristics within established safety profiles, vaccination was often viewed as protective rather than risky. Conversely, unresolved uncertainty amplified caution and, in some cases, delayed or foreclosed decision-making (4). Together, these patterns indicate that safety-benefit appraisal in non-NIPV decisions is contingent, sequential, and highly sensitive to the quality of explanation.

### Interpretation of theme 3: economic considerations

Findings from Theme 3 demonstrate that economic considerations in non-NIPV decision-making extend beyond simple affordability. Mothers did not uniformly frame cost as opposition to vaccination; rather, financial reasoning operated through differentiated logics shaped by household resources, perceived value, and competing priorities. For some families, cost functioned as an initial filter that determined whether non-NIPV entered consideration at all, while for others it became a decisive barrier only after safety and benefit had been acknowledged, similar as other studies (12).

Several mothers articulated an investment-oriented perspective, evaluating vaccination in terms of anticipated reductions in future medical expenses, caregiving burden, and emotional stress (13). This reasoning was often accompanied by selective prioritization, whereby certain vaccines were deemed worth paying for while others were deferred or declined. Such patterns suggest that mothers engage in implicit cost-effectiveness reasoning, even in the absence of formal economic frameworks.

Importantly, economic hesitation was frequently intertwined with perceptions of institutional endorsement (14). Questions regarding why vaccines considered important for child health required full out-of-pocket payment reflected not only financial concern, but also uncertainty about policy positioning and responsibility allocation (15). In this sense, cost considerations functioned as a proxy for broader judgments about safety, effectiveness, and perceived necessity, rather than operating as an isolated financial constraint (16).

### Interpretation of theme 4: trust and healthcare communication

Theme 4 highlights that trust in the context of non-NIPV is constructed through concrete communication encounters rather than assumed as a stable attribute of the healthcare system (17). Mothers consistently emphasized the importance of clear explanations, patience, and responsiveness in shaping their confidence in vaccination decisions. Communication that addressed not only benefits but also potential adverse reactions and post-vaccination management was described as particularly reassuring.

However, many mothers experienced routine vaccination encounters as time-constrained and transactional, especially when non-NIPV were introduced briefly as optional add-ons. Such interactions did not necessarily erode trust in professional authority, but they limited opportunities for clarification and left questions unresolved. As a result, mothers often turned to informal information sources after clinical encounters, increasing exposure to inconsistent or emotionally charged narratives.

Transparency emerged as a key element in maintaining credibility. Mothers expressed greater trust when healthcare providers openly acknowledged uncertainty and avoided overly directive recommendations. These findings suggest that trust in non-NIPV decision-making is not solely dependent on professional expertise, but on whether communication aligns with mothers’ informational and emotional needs within constrained clinical settings (18).

### Interpretation of theme 5: system-level experience

Theme 5 underscores the influence of system-level service experiences on maternal engagement with non-NIPV decisions. Procedural convenience, waiting times, and clinic organization were described as shaping not only logistical feasibility but also cognitive and emotional readiness to consider additional vaccines. When vaccination services were perceived as orderly and child-centered, mothers reported greater willingness to engage with discretionary decisions (5, 19).

At the same time, digitalization of vaccination services was generally perceived as desirable, particularly for improving efficiency in appointment scheduling and record management. Mothers expressed a clear preference for more convenient and user-friendly digital platforms that could facilitate access to vaccination services.

Across interviews, mothers expressed a desire for greater continuity in vaccination-related support. Non-NIPV decisions often involve delayed reflection or require reassurance across multiple doses, yet existing services were experienced as episodic and fragmented. The absence of clear follow-up channels reinforced reliance on informal networks and contributed to prolonged hesitation. Taken together, features of vaccination service organization, particularly the availability of follow-up communication and continuity beyond single clinic visits, appear to facilitate or constrain non-NIPV uptake by shaping mothers’ sense of support during the decision-making process.

The relationship between the present findings and previous qualitative literature is summarized in Table 4.

**Table 4:** Comparison of the present findings with previous qualitative evidence and contribution of the current study.

Discussion point	Relationship with previous qualitative literature	Contribution of the present study
Multiple sources of vaccination information	Consistent with previous studies, parents relied on both healthcare professionals and informal information sources, while experiencing uncertainty regarding information quality.	Extends previous evidence by demonstrating how mothers actively verify information across multiple channels and how information asymmetry influences confidence in non-NIPV decision-making.
Safety benefit trade-offs in vaccination decision-making	Previous studies have similarly identified vaccine safety as the primary determinant of parental vaccination decisions.	Further illustrates the dynamic process by which mothers balance perceived vaccine safety against disease severity, preventive effectiveness, and child-specific health characteristics.
Tension between economic burden and health investment	Finding of the presented study aligns with earlier evidence that vaccine cost is an important barrier to non-NIPV uptake.	Demonstrates that mothers simultaneously evaluate affordability, long-term health benefits, and perceived return on investment, rather than considering price alone.
Trust building and gaps in healthcare communication	Existing qualitative literature has consistently emphasized the importance of healthcare professionals in parental vaccine decision-making.	Provides more detailed evidence regarding how communication quality, consultation time, empathy, and transparency shape trust in vaccination recommendations.
System-level support and individual expectations in service experience	Previous studies have highlighted the need for improved vaccination services and policy support.	Expands existing knowledge by identifying unmet needs related to digital vaccination services, follow-up support, and more person-centered service delivery.

### Limitations

Several limitations should be acknowledged. First, this study was conducted at a single center, and the findings may reflect context-specific characteristics of the local healthcare system and population. Accordingly, the transferability of these findings to other healthcare settings, geographic regions, or populations should be interpreted with caution. Second, although the sample size was limited, interviews were continued until thematic saturation was achieved, consistent with qualitative research principles. Nevertheless, some less common perspectives or context-specific experiences may not have been fully captured. Third, participants were recruited from mothers who had previously participated in a questionnaire survey, which may introduce selection bias, as these participants may have been more willing to engage in research or discuss vaccination-related issues than mothers who declined participation. Consequently, the findings may overrepresent these perspectives and underrepresent views from less engaged mothers. Finally, as with all interview-based studies, responses may have been influenced by social desirability or recall bias. Accordingly, participants may have underreported uncertainty, hesitation, or socially undesirable views regarding vaccination decisions.

## Summary and conclusion

This qualitative study shows that maternal decision-making regarding non-NIPV is a dynamic and context-dependent process, rather than a reflection of fixed attitudes. Across the five themes, decisions were shaped by information uncertainty, safety thresholds, economic considerations, communication experiences, and system-level service conditions. Family context, while not the sole determinant of vaccination outcomes, influenced mothers’ sensitivities and priorities across these domains, thereby shaping how non-NIPV decisions were ultimately negotiated.

## Data Availability

The original contributions presented in the study are included in the article/supplementary material, further inquiries can be directed to the corresponding authors.
